# Supplementary material for: Development of a computer-aided design software for the quantitative evaluation of aesthetic damage
Source: PLoS One. 2019 Dec 18;14(12):e0226322. doi: 10.1371/journal.pone.0226322 (PMC6919621; doi:10.1371/journal.pone.0226322)
Supplement: S1 Code site — 1. (ZIP) [file pone.0226322.s001.zip › [EESC_jr][Projeto]Relatorio_07_21/vendors/select2/docs/_includes/options/dropdown/placement.html]

## Can I change how the dropdown is placed?

### Can the dropdown be placed directly after the selection container?

{% include options/not-written.html %}

### Can I pick an element for the dropdown to be appended to?

{% highlight js linenos %}
$('select').select2({
dropdownParent: $('#my\_amazing\_modal')
});
{% endhighlight %}
{% include options/not-written.html %}

### I'm using a Bootstrap modal and I can't use the search box

Use the `dropdownParent` option, setting it to the modal.

{% include options/not-written.html %}

### I'm using jQuery UI and I can't use the search box

{% include options/not-written.html %}
